# Prehospital Administration of Antibiotics for Patients with Suspected Severe Infection: A Systematic Review and Meta Analysis

**DOI:** 10.3390/antibiotics15090855

**Published:** 2026-09-01

**Authors:** Ayman El-Menyar, Ibrahim Al-Hassani, Mashhood Naduvilekandy, Rafael Consunji, Fernando Netto, Airton Leonardo de Oliveira Manoel, Prabath Nanayakkara, Sagar Galwankar, Łukasz Szarpak, Basar Cander, Eman Elmenyar, Sandro Rizoli, Hassan Al-Thani

**Affiliations:** 1 Department of Surgery, Trauma Surgery, Clinical Research, Hamad Medical Corporation, Doha 3050, Qatar; v-mnaduvilekandy@hamad.qa; 2 Department of Clinical Medicine, Weill Cornell Medical College, Doha P.O. Box 24144, Qatar; 3 Department of Medical Education, Weill Cornell Medical College, Doha 24144, Qatar; iaa4004@qatar-med.cornell.edu; 4 Department of Surgery, Trauma Surgery, Hamad Medical Corporation, Doha P.O. Box 3050, Qatar; rconsunji@hamad.qa (R.C.); fnetto@hamad.qa (F.N.); amanoel@hamad.qa (A.L.d.O.M.); srizoli@hamad.qa (S.R.); halthani@hamad.qa (H.A.-T.); 5 General Internal Medicine, Amsterdam Public Health Research Institute, Amsterdam University Medical Centers, 1081 HV Amsterdam, The Netherlands; p.nanayakkara@amsterdamumc.nl; 6 Department of Emergency, Emergency Medicine Residency Program, Florida State University College of Medicine, Sarasota Memorial Hospital, Sarasota, FL 34243, USA; sagar.galwankar@med.fsu.edu; 7 Institute of Medical Sciences, The John Paul II Catholic University of Lublin, 20-950 Lublin, Poland; lukasz.szarpak@kul.pl; 8 Department of Emergency Medicine, Bezmialem Vakif University, Fatih, Istanbul 34093, Turkey; basar.cander@bezmialem.edu.tr; 9 Faculty of Medicine, Bahcesehir University, Istanbul 34734, Turkey; eelmenyar@gmail.com

**Keywords:** sepsis, prehospital care, emergency medical services, early antibiotic therapy, septic shock, systematic review, meta-analysis

## Abstract

**Background**: Sepsis is a time-sensitive, serious condition; therefore, early recognition and prompt initiation of therapy are critical to improving outcomes in patients with infection. We sought to evaluate whether prehospital antibiotic administration (PHAA) is associated with improved outcomes in patients with suspected severe sepsis. **Methods**: We conducted a systematic review and meta-analysis. This review was conducted in accordance with the PRISMA guidelines, and the protocol was developed a priori and registered with the International Prospective Register of Systematic Reviews (PROSPERO). PubMed, Embase, Cochrane, and Scopus were searched from inception through January 2026. Randomized controlled trials (RCTs) and observational studies evaluating empirical PHAA compared with the standard of care were included. The risk of bias and certainty of evidence were assessed. Outcomes included short- and long-term mortality, hospital length of stay (HLOS), and intensive care unit (ICU) admission. Sensitivity analyses were performed. **Results**: Eleven studies recruiting 23,146 patients were included (three RCTs and eight observational studies). Restricting the short-term mortality analysis to RCTs alone showed no mortality benefit with PHAA (RR 0.96, 95% CI 0.74–1.23), whereas the broader, observational-dominated pooled analysis suggested a possible reduction in short-term mortality (RR 0.84, 95% CI 0.68–1.02, I^2^ = 51.0%), reaching statistical significance when restricted to the adult sepsis/septic shock population (RR 0.82, 95% CI 0.69–0.99, I^2^ = 18.5%). No significant difference in short-term mortality was observed in the exploratory subgroup analysis of two high-severity studies (RR 0.89, 95% CI 0.67–1.18). There were no significant long-term mortality differences in the observational and RCT studies (RR 0.86, 95% CI 0.71–1.04 and RR 1.09, 95% CI 0.76–1.54, respectively). Patients who received PHAA had no significantly different ICU admissions (RR 1.12, 95% CI 0.95–1.33) or HLOS (MD −1.79 days, 95% CI −6.05 to 2.47). Even though individual studies showed no significant difference, pooled HLOS was significantly higher with PHAA for the RCT subgroup (MD 0.66, 95% CI 0.42–0.90, I^2^ = 0.00%) after conversion of median to mean values (for better statistical compatibility). **Conclusions**: PHAA was not associated with a significant reduction in short-term mortality, though a significant reduction was observed when restricted to adult sepsis/septic shock population. No mortality benefit was found in pooled RCTs, mixed-age population studies, or long-term mortality. Observational studies suggest a possible mortality benefit, whereas randomized evidence does not confirm it. This discrepancy may reflect confounding by indication, patient selection, severity, and differences in EMS systems rather than a true treatment effect. The certainty of evidence remains very low, and data on prehospital antibiotic type, blood culture, and infection severity are lacking. Therefore, high-quality prospective RCTs are warranted.

## 1. Introduction

Despite recent medical advances, sepsis remains a major global cause of death [1,2]. According to the current Sepsis-3 consensus [3], sepsis is defined as a life-threatening organ dysfunction caused by a dysregulated host response to infection, which can rapidly progress [4,5,6]. Sepsis is a time-sensitive, serious condition; therefore, early recognition and prompt initiation of appropriate therapy are critical to improving outcomes in patients with suspected sepsis. The Surviving Sepsis Campaign (SSC) guidelines 2021 recommend administering antimicrobials within 3 h of recognition of sepsis [7]. However, prior data did not consistently demonstrate a clear reduction in mortality with earlier antibiotic administration in patients without shock [7,8,9,10]. Data from three Dutch hospitals showed that a reduction in the time to antibiotic administration was not associated with better outcomes, regardless of the degree of sepsis [8]. This highlights an important discrepancy between the urgency emphasized in clinical practice guidelines and the relatively low certainty of supporting evidence. In addition, concerns have been raised that prioritizing rapid antimicrobial administration may contribute to inappropriate antibiotic use and antimicrobial resistance [4]. Furthermore, timely administration may not be feasible in all patients with sepsis, particularly when diagnostic uncertainty is present [11,12].

A few studies revealed that delayed administration of antibiotics led to worse outcomes, and each hour of delay was associated with a 7.6% increase in mortality [13]. Moreover, the mortality benefit and less progression to septic shock with early antibiotics was observed in patients with severe sepsis and with less progression to septic shock [14,15]. In this regard, the odds of mortality increased within each sepsis severity stratum, particularly in septic shock [15]. To improve the time to deliver healthcare service appropriately across the system, Alam et al. (PHANTASi trial) concluded that the emergency medical services (EMS) personnel should be trained to diagnose and treat sepsis on time [16]. However, they did not recommend initiating prehospital antibiotics.

Despite these concerns, studies on the feasibility and effectiveness of prehospital antibiotic administration (PHAA) for patients with suspected infection, suspected sepsis, and traumatic open wounds remain insufficient or inconclusive. Yet a critical question remains: does PHAA reduce mortality, progression of severity, ICU admissions, or ICU length of stay? Notably, detecting the type and severity of infection is not widely feasible in prehospital settings [17,18]. Moreover, the evidence for any time-gain benefit from PHAA is unclear [18]. The current evidence regarding feasibility, safety, and mortality benefit from PHAA remains inconsistent [16,19].

In practice, PHAA is positioned at the intersection of two competing priorities: time-critical treatment for life-threatening infection and diagnostic uncertainty in the field. Any evaluation of this strategy must therefore consider not only time gain, but also case selection, illness severity, microbiological consequences, and the risk of unnecessary antimicrobial exposure. Several studies addressed related but narrower questions, such as Varney et al. [20] and Poynter et al. [21], who evaluated PHAA in a sepsis-only population with an earlier search cutoff (through 2021–2023) and fewer studies numbers (4–5); and a Cochrane review of meningococcal disease [22] identified only one eligible RCT.

To address these gaps, we performed a systematic review and meta-analysis to evaluate the hypothesis that PHAA is associated with improved outcomes across medical populations, including sepsis/septic shock and invasive meningococcal disease (IMD) and trauma patients receiving prophylactic antibiotics, as well as reduced ICU admissions and hospital length of stay (HLOS). Because antibiotics are administered before a diagnosis is confirmed in each of these settings, they were treated as related prehospital-decision scenarios rather than a single disease question, with trauma and IMD analyzed separately from the general sepsis cohort throughout. By translating these findings into EMS, our study could provide healthcare providers and decision-makers with evidence and updates on PHAA practice.

## 2. Results

### 2.1. Study Selection and Design

**Figure 1:**
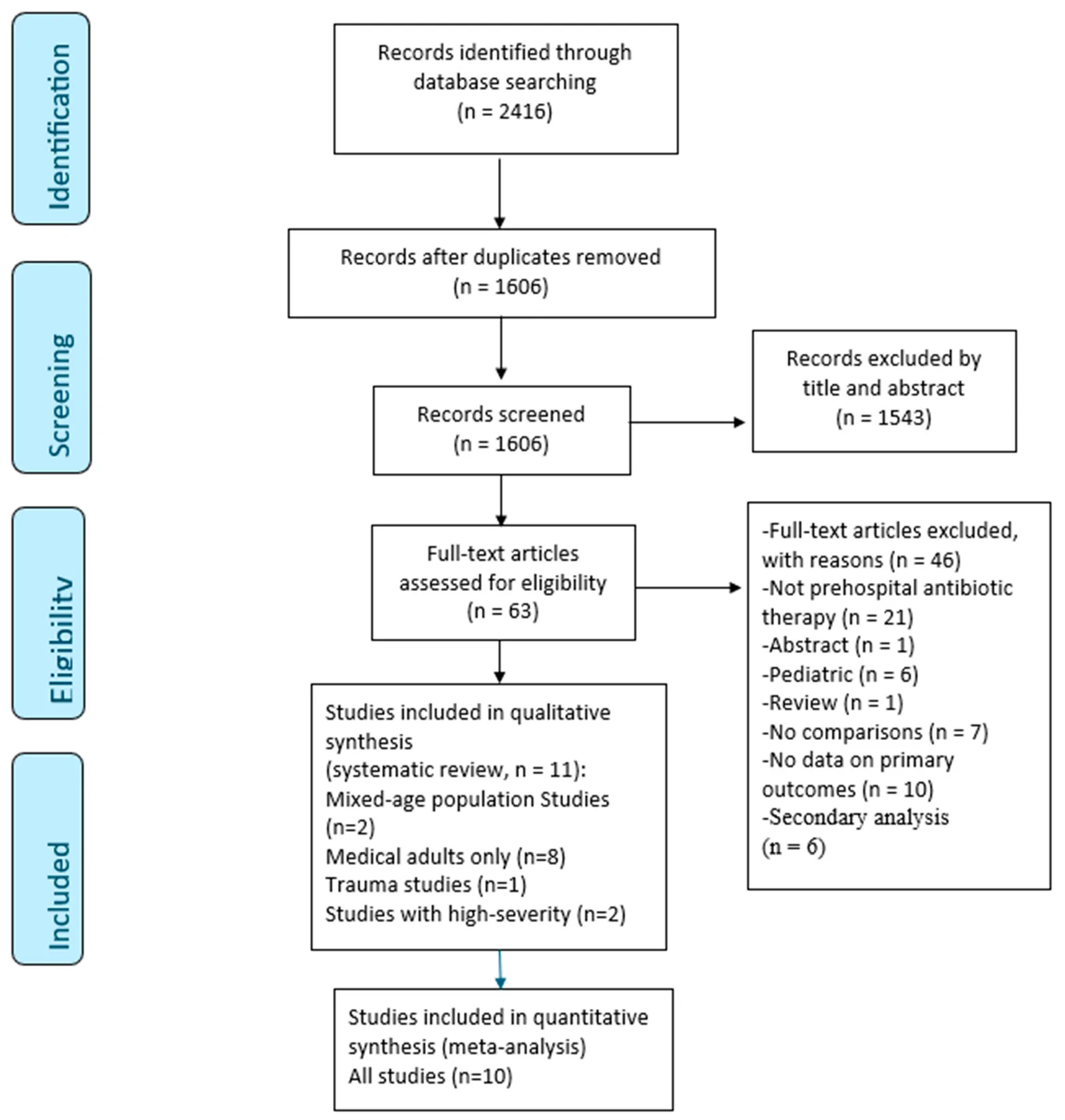
PRISMA flowchart for prehospital antibiotic administration versus usual care.

A total of eleven studies met the inclusion criteria and were included in the systematic review (Figure 1). Ten studies evaluated medical indications such as sepsis, septic shock, and IMD, whereas one study [23] evaluated trauma patients receiving prophylactic antibiotics for open wounds (Table 1). Three studies were RCTs [16,24,25], and eight were observational cohort or registry-based studies [19,23,26,27,28,29,30,31]. Ascertainment of comparator (non-PHAA) status varied by study design: in the randomized trials, the comparator arm was defined by treatment allocation; in observational and registry-based studies, comparator patients were identified from the absence of a documented antibiotic administration in prehospital or hospital records; and in three studies employing a pre–post-implementation design [19,28,30], the comparator consisted of a historical cohort treated prior to protocol implementation rather than a concurrent no-antibiotic group. Across all the studies, a total of 23,146 patients were analyzed, of whom 18,276 (79%) were derived from the single registry-based trauma study (combat victims) [23]. In this trauma study, 13% of patients (n = 2384) received prehospital antibiotics in accordance with Tactical Combat Casualty Care recommendations.

### 2.2. Study Populations and Clinical Context

Illness severity ranged from suspected infection and uncomplicated sepsis to septic shock. Two studies enrolled patients with clearly high-acuity illness, including those with septic shock or hemodynamic instability sepsis [27,31], while Jones et al. targeted patients with suspected severe sepsis based on prehospital screening criteria [25]. Two studies evaluated IMD [26,29]. The mean age of IMD patients was 19 years. These studies were initially retained for their relevance to PHAA in rapid progressive infection; however, because of their mixed-age population, a sensitivity analysis was performed, excluding them. In the two IMD studies, the decision to give antibiotics was driven by recognizable clinical features, as there was no point-of-care diagnostic test available at the prehospital setting.

### 2.3. Prehospital Antibiotic and Regimen

Across medical studies, antibiotics were predominantly administered intravenously, with ceftriaxone 2 g IV being the most frequently reported agent in randomized and feasibility trials [16,24]. Observational sepsis cohorts reported a range of empiric broad-spectrum antibiotic regimens consistent with local EMS protocols, including third-generation cephalosporins and penicillin-based β-lactam therapies [16,19,24,27,28,30,31]. In studies evaluating IMD, both oral and parenteral antibiotics were reported [26,29]. Oral antibiotics were more commonly administered to patients with less severe presentations [26].

In the trauma study, PHAA was used for prophylaxis, and was predominantly with β-lactam agents [23]. A subgroup analysis by antibiotic type was not performed, because of mixed antibiotic regimens, which did not provide stratified data, precluding meaningful comparisons.

### 2.4. Clinical Outcomes

Three RCTs reported mortality outcomes at different time points, and none demonstrated a statistically significant mortality benefit associated with PHAA. In contrast, an association with lower short-term mortality emerged in several observational studies, particularly those focusing on patients with sepsis or septic shock (Table 1). In IMD, outcomes varied with disease severity and route of antibiotic administration. Parenteral PHAA was associated with higher mortality [24]. This association was particularly pronounced among patients with severe disease manifestations [29]. In contrast, the other IMD cohort [26], PHAA, was associated with lower unadjusted in-hospital mortality. Although this association was significant in the multivariable analysis, it was not statistically significant after propensity-score adjustment.

**Trauma setting**: In the large combat trauma cohort of patients with open wounds [23], PHAA was associated with lower unadjusted in-hospital mortality compared with no PHAA (2.6% vs. 4.0%), based on survival-to-hospital-discharge data.

**Figure 2:**
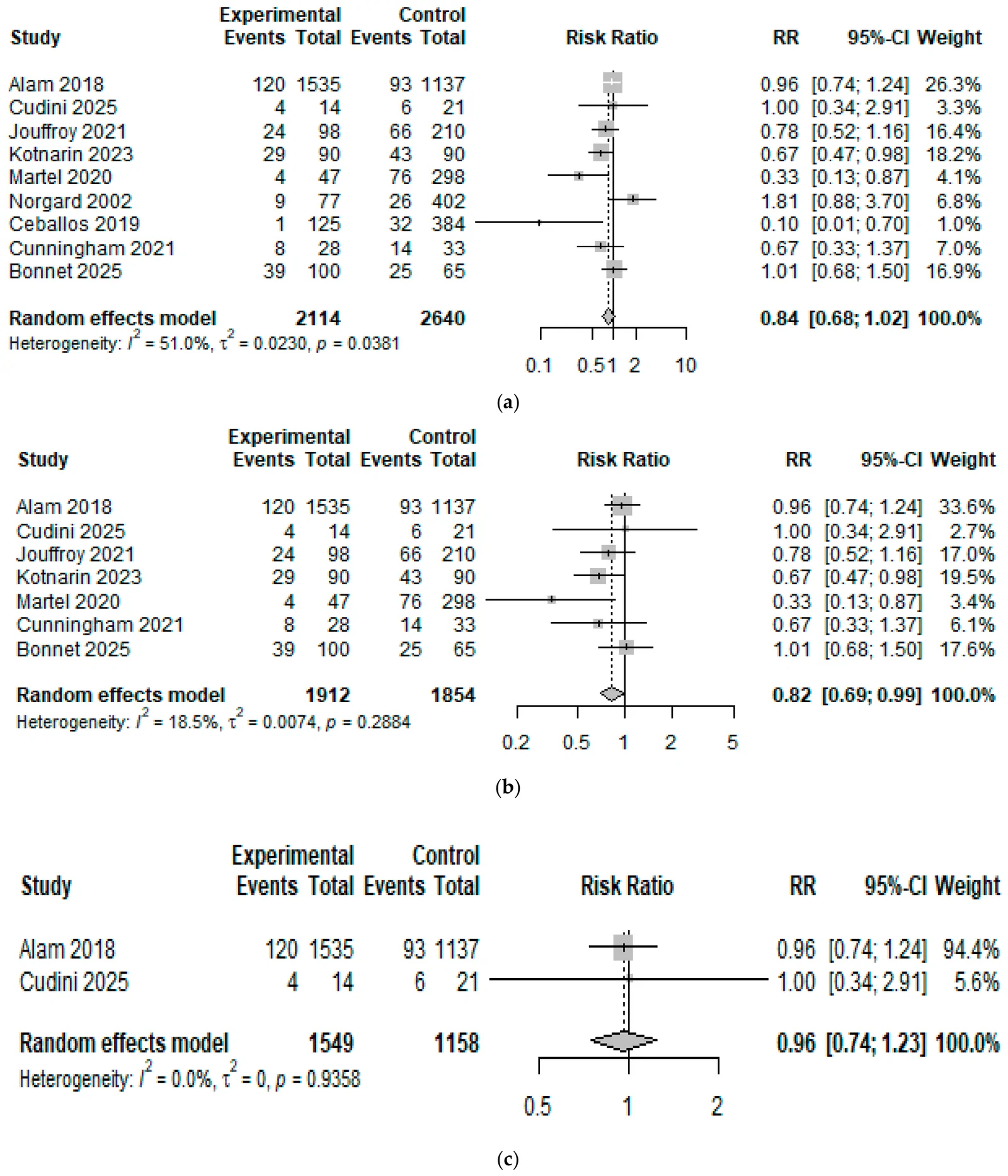
(**a**) Forest plot for 9 studies [16,19,24,26,27,28,29,30,31] reporting short-term mortality for medical case only; (**b**) forest plot for short-term mortality for sepsis/septic shock cases only [16,19,24,27,28,30,31]; and (**c**) forest plot for short-term mortality for sepsis/septic shock cases for RCT only [16,24].

**Meta-analyses:** The study-level findings were further explored through quantitative synthesis. Pooling of medical cohort studies (nine studies) showed no statistically significant reduction in short-term mortality (RR 0.84, 95% CI 0.68–1.02) (Figure 2a).

We planned to examine the mixed-age/pediatric exclusion and the IMD exclusion as two separate sensitivity analyses; however, both excluded the same two studies [26,29], yielding an identical result. As this overlap is coincidental to the current dataset rather than a conceptual equivalence, we refer to this population throughout as the adult sepsis/septic shock population, reflecting both exclusions simultaneously. Restricting the medical-cohort pooling to this population (seven studies) demonstrated a statistically significant reduction in mortality (RR 0.82, 95% CI 0.69–0.99) (Figure 2b). This finding and the preceding nine-study estimate were dominated by observational data (RCTs contributed under 25% of the pooled weight); restricting the same short-term mortality outcome to the two contributing RCTs [16,24] showed no mortality benefit (RR 0.96, 95% CI 0.74–1.23) (Figure 2c).

**Figure 3:**
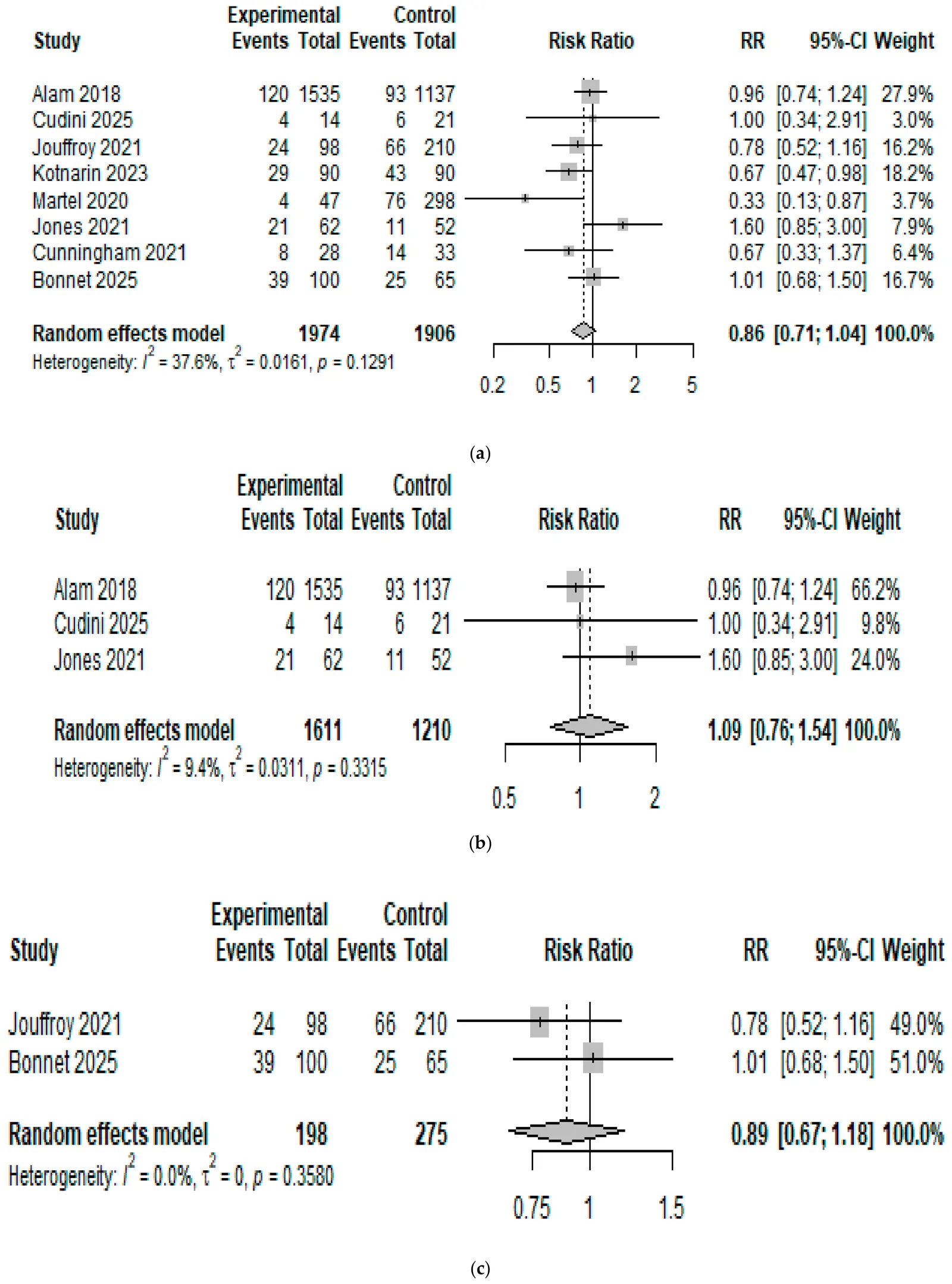
(**a**) Forest plot for 8 studies [16,19,24,25,27,28,30,31] reporting total mortality (short- and long-term) for sepsis/septic shock case only; (**b**) total mortality (short- and long-term) for RCT only [16,24,25]; and (**c**) forest plot for 2 studies [27,31] with severe sepsis (short-term mortality).

The point estimate remained in the same direction, though the pooled estimate did not reach statistical significance when short- and long-term mortality were pooled across eight studies (RR 0.86, 95% CI 0.71–1.04). (Figure 3a). Analyses restricted to RCTs showed no statistically significant mortality benefit (RR 1.09, 95% CI 0.76–1.54) (Figure 3b). Subgroup analysis including only two high-severity studies defined as septic shock or hemodynamic instability [27,31] did not show a significant short-term mortality difference (RR 0.89, 95% CI 0.67–1.18); given the small number of studies and wide confidence interval, this finding should be interpreted as exploratory and hypothesis-generating rather than conclusive (Figure 3c).

### 2.5. ICU Admission and Length of Stay

**Figure 4:**
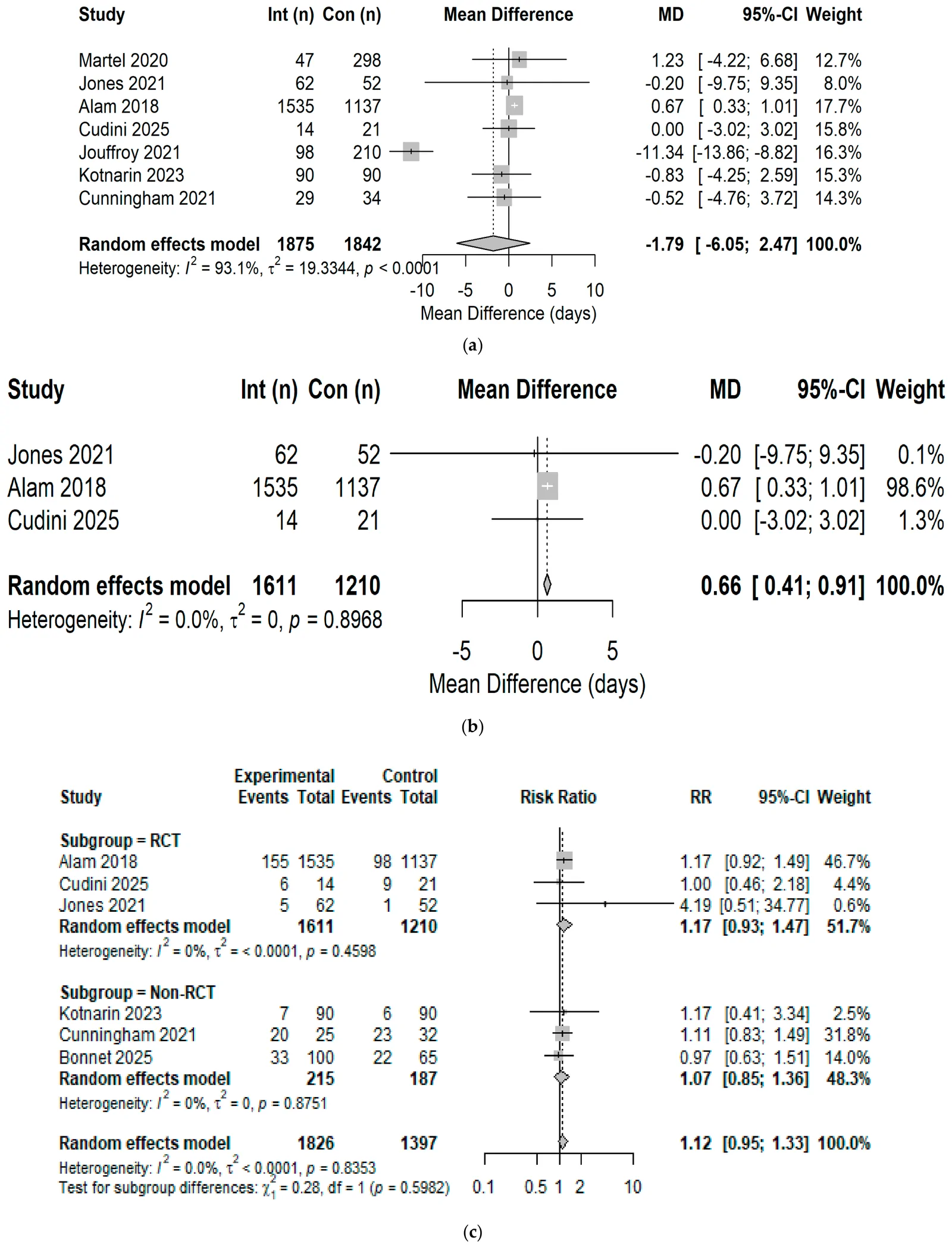
(**a**) Forest plot for hospital length of stay from 7 adult sepsis/septic shock studies [16,19,24,25,27,28,30]; (**b**) forest plot for HLOS from 3 RCTs [16,24,25]; and (**c**) forest plot for ICU admission from 6 studies [16,19,24,25,30,31], along with subgroup analysis.

Among the randomized and feasibility trials, no significant difference in HLOS was observed between patients who received prehospital antibiotics and those who received standard care. Jouffroy et al. [27] was the only observational study to report a statistically significant difference, with a shorter HLOS in the PHAA group (7 vs. 17 days). A meta-analysis pooling seven studies showed no significant overall difference in the HLOS (Figure 4a).

In subgroup analysis restricted to RCTs, the pooled estimate suggested a small difference of 0.6 days (Figure 4b), largely driven (99%) by the PHANTASi trial [16]. This apparent significance arose from the conversion of the PHANTASi trial’s median/IQR HLOS values to mean/SD meta-analytic pooling; the original trial report found no statistically significant difference in HLOS between groups. This RCT-only estimate should therefore be interpreted with caution (mean difference is for better statistical compatibility).

Among studies reporting ICU LOS, only Martel et al. [28] demonstrated a statistically significant difference, with a longer ICU stay in the control group (5.18 days vs. 2.51 days). The other studies reported no statistically significant differences in ICU LOS between intervention and control groups [16,19,27,30,31]. Six studies reported data on ICU admission [16,19,24,25,30,31], and none demonstrated a statistically significant difference between patients receiving prehospital antibiotics and those receiving standard care (Figure 4c). Given the potential for confounding by indication in observational data, we performed a subgroup analysis by study design: RCTs alone showed no significant difference (RR 1.17, 95% CI 0.93–1.47), nor did observational studies alone (RR 1.07, 95% CI 0.85–1.36); and there was no statistical evidence that the two subgroups differed (*p* = 0.60 for subgroup interaction) (Figure 4c).

### 2.6. Time to PHAA

Only a subset of studies reported quantitative time-to-antibiotic data, and the reported intervals represented different phases of patient care. Alam et al. [16] (PHANTASi trial) reported antibiotic timing relative to ED arrival (26 min before ED arrival vs. 70 min after ED arrival); Cudini et al. [24] (PASS trial) measured time from paramedic scene arrival to antibiotic administration (42 vs. 150 min); Cunningham et al. [30] reported time from 9-1-1 call receipt to antibiotic administration (36.04 vs. 220.7 min); and Kotnarin et al. [19] reported prehospital EMS operational timing without a clearly defined temporal anchor (16.0 ± 7.4 vs. 50.9 ± 29.4 min). Furthermore, transport duration and exact timing from antibiotic administration to ED arrival were not consistently available across studies, limiting direct comparability of these operational intervals. A meta-analysis of time to antibiotic administration could not be performed, because these studies used different reference points along the care pathway, making direct comparisons across studies unreliable. These reference points are summarized in Appendix A Table A1.

### 2.7. Blood Culture

Blood-culture data were variably reported across studies. In the PHANTASi randomized trial [16], blood cultures were positive in 389 patients (35%) in the PHAA group compared with 279 patients (26%) in the usual care group (*p* < 0.0001). In the PASS feasibility trial [24], prehospital blood cultures were obtained in 31 of 35 patients (89%), of which 13 (42%) yielded bacterial growth; however, no comparative analysis by antibiotic exposure was reported. Cabellos et al. [26] reported blood-culture positivity in 201 of 384 patients (52%) who did not receive PHAA, compared with 7 of 125 patients (6%) who did receive PHAA. However, the timing of blood-culture collection relative to antibiotic administration was not reported. Other studies reported microbiological growth in blood or cerebrospinal fluid primarily for diagnostic case identification and did not compare culture positivity by antibiotic exposure.

### 2.8. Quality of Studies and GRADE

Study quality varied across the included studies. Among the RCTs, one was judged to be at low risk of bias, whereas two were judged to be at high risk due to limitations in allocation concealment, early termination, or selective reporting (Appendix A Table A2). Observational studies were generally of moderate methodological quality according to the NOS, with common limitations including residual confounding, the use of historical controls, and incomplete follow-up reporting (Table 2). Using the GRADE approach, the certainty of evidence for all outcomes was rated as very low. This rating was primarily driven by the limited contribution of RCTs (less than 25% of the pooled weight, which did not determine the direction of effect, as well as further downgrading for risk of bias, inconsistency, and imprecision.

### 2.9. Publication Bias

**Figure 5:**
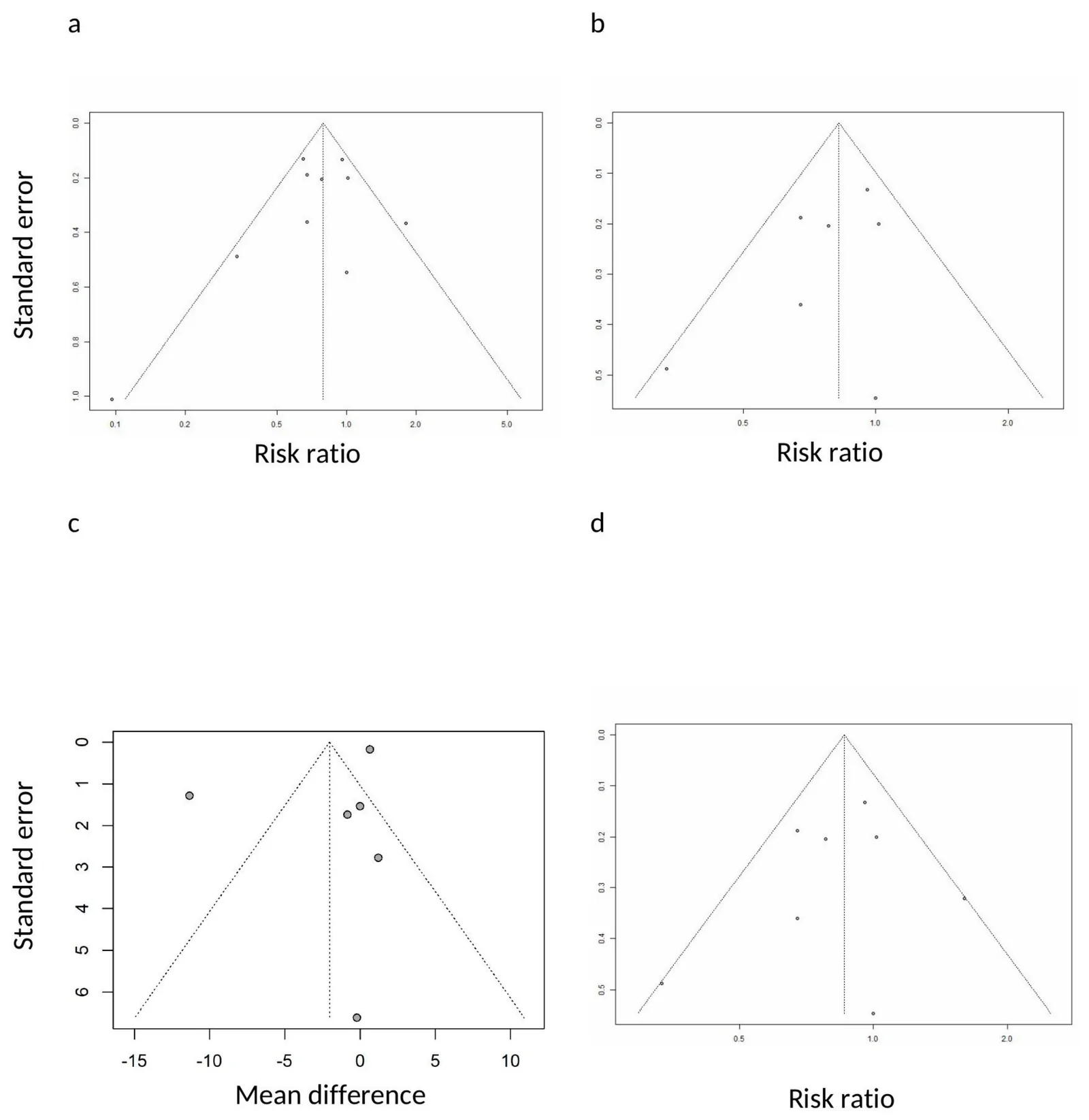
Publication bias: (**a**) Funnel plot for short-term mortality (Egger’s *p*-value 0.53); (**b**) funnel plot for sepsis/septic shock short-term mortality (Egger’s *p*-value 0.21); (**c**) funnel plot for hospital length of stay for sepsis/septic shock (Egger’s *p*-value 0.78); and (**d**) funnel plot for sepsis/septic shock total mortality (Egger’s *p*-value 0.59).

Funnel plots and Egger’s test results are presented in Figure 5 and Appendix A Figures. There was no statistically significant evidence of publication bias or small-study effects in the analyzed studies, as Egger’s test *p*-values were >0.05. However, publication bias should be cautiously considered if the analyzed studies were <10.

### 2.10. Funding Source

The funding source was reported explicitly only in the PHANTASi randomized trial [16], which received public, non-commercial funding. The remaining studies did not report funding information, limiting the assessment of potential funding-related bias.

## 3. Discussion

This systematic review evaluated whether administering antibiotics before hospital arrival, which is not yet standard care, improves clinical outcomes in patients with suspected severe sepsis. A pooled analysis showed that PHAA was associated with reduced short-term mortality. However, this mortality benefit was driven primarily by observational studies rather than RCTs. This discordance between null randomized evidence and a more favorable observational signal was consistent across mortality analyses and may reflect confounding by indication, patient selection, or differences in EMS systems rather than a true treatment effect.

The role of early antibiotic therapy in the treatment of infections and sepsis has been debated for decades [7,8,9,10,13]. The divergence between randomized and observational evidence is clinically important. Whereas RCTs did not demonstrate a mortality signal, several observational cohorts suggested benefit, particularly in higher-acuity populations. Such a pattern may reflect a true effect in selected phenotypes, but it may also reflect residual confounding, differences in system organization, or other co-interventions associated with advanced prehospital care. Guidelines emphasize early antibiotics in suspected sepsis, particularly in patients with septic shock [7], yet no major guideline recommends routine PHAA [7,17]. However, the clinically distinct use-cases for PHAA are in favor of empiric treatment for suspected sepsis, early treatment of rapidly progressive invasive bacterial disease, and contaminated trauma. This distinction is essential when interpreting pooled estimates, as the indication for antibiotic administration, baseline mortality risk, and likelihood of confounding differ substantially across these groups. Therefore, the studies included in this analysis encompassed heterogeneous clinical populations.

Moreover, RCTs such as PHANTASi [16] enrolled patients with suspected infection rather than confirmed sepsis, and PHAA was within relatively short time frames after hospital arrival. Under these circumstances, the additional benefit of PHAA may be difficult to detect.

Although the reported data on the time to PHAA are inconsistent, these findings demonstrate that PHAA is operationally feasible within EMS systems and can meaningfully accelerate the administration of antimicrobial therapy. However, the clinical relevance of this time advantage remains uncertain. Prior studies have suggested that the survival benefit associated with earlier antibiotic administration may be most pronounced in patients with septic shock. However, our analysis did not show a significant difference in mortality benefit in severe sepsis [27,31].

In the study by Nørgård et al., in Denmark [29], parenteral PHAA was associated with higher mortality in IMD, but this likely reflected confounding by indication, as such treatment was reserved for patients with more severe clinical features. The predictors of mortality were age and shock. In contrast, Cabellos et al. [26], in Spain, reported lower unadjusted mortality following IMD in the PHAA group; however, the authors attributed this to the quality of hospital care and the infection serogroup.

The mortality differences following PHAA highlight the complexity of interpreting pooled estimates across heterogeneous populations. Nonetheless, sensitivity analyses restricted to adult sepsis/septic shock population yielded results consistent with the primary findings, suggesting that the overall mortality signal was not driven solely by these distinct populations. Given its borderline confidence interval and the null RCT-only finding, this result should be regarded as hypothesis-generating rather than conclusive.

A further issue is whether host factors, including age, may modify the effect of PHAA. Although this meta-analysis was not designed to formally evaluate effect modification, age-related differences merit consideration. Subgroup findings from the PHANTASi trial have suggested that younger patients may derive greater benefit from PHAA [16]. At present, the available evidence is insufficient to conclude that age meaningfully modifies the treatment effect, but it does support further prospective investigation. Host age acts as a critical effect modifier in prehospital antibiotic outcomes because physiological aging directly alters baseline bacteremia kinetics, pathogen diversity, and drug clearance [32,33,34].

Blood-culture findings were inconsistently reported, precluding firm conclusions, but they raise an important methodological issue. In PHANTASi, blood-culture positivity was higher in the PHAA group [16], whereas in the Cabellos cohort, the opposite pattern was observed [26]. These discrepant findings may reflect differences in the timing of specimen collection, disease severity, or diagnostic workflow. Future studies should standardize blood-culture collection and report these data more transparently, as culture positivity may influence both diagnostic certainty and downstream management.

Safety outcomes were poorly reported overall. Randomized and feasibility trials that prospectively monitored adverse events did not identify major safety concerns, with only rare mild allergic reactions reported [16]. As a result, the current evidence is insufficient to confidently define the safety profile of routine PHAA, particularly regarding allergic reactions, inappropriate treatment, and unintended antimicrobial exposure in patients ultimately found not to have bacterial infection.

There is no consistent evidence on the impact of PHAA on HLOS and ICU admission. This may indicate that, while PHAA accelerates treatment initiation, its downstream effects on resource utilization are limited or overshadowed by other determinants of hospital course, including baseline severity of illness, organ dysfunction, source control, and local discharge practices. The present meta-analysis of RCTs showed a shorter HLOS in patients with PHAA [16,24,25].

The favorable observational signal reflects a systems-of-care effect rather than a pure drug-timing effect [20,35,36]. The EMS teams could be capable of sepsis recognition, obtaining cultures, activating appropriate destination pathways, and delivering protocolized treatment, which may achieve better outcomes for multiple reasons that go beyond antibiotic administration alone [10]. In such contexts, PHAA may serve as a marker of organized, high-performance prehospital care rather than as an isolated causal intervention [16]. This is where antimicrobial stewardship becomes central. At the ED, diagnostic uncertainty can be partially reduced with laboratory testing, imaging, serial reassessment, and early specialist input [7]. However, in the prehospital settings, these safeguards are inherently limited [36]. Therefore, protocols that bring antibiotics into the prehospital setting must also specify who should not receive them, how microbiological sampling will be performed, and how the appropriateness of treatment will be audited after hospital arrival [7]. Moreover, whether the EMS staff (paramedic- or physician-based service) are authorized and trained to initiate empiric IV antibiotics and collect swabs or blood samples before hospital arrival?

As almost 50% of hospitalized patients arrive at the hospital via EMS, the potential benefit of PHAA is most plausible in patients with a likelihood of bacterial infection, significant physiological derangement, and a meaningful anticipated delay to in-hospital treatment [10,16,35,37]. Conversely, in lower-acuity presentations or in systems where hospital antibiotics are administered promptly upon arrival, the added benefit of prehospital dosing is likely marginal [7,10,16,36].

In the medical settings, the EMS could easily collect information to support giving PHAA as a possible therapeutic option. However, in a trauma setting, the absence of clinical and laboratory indicators apart from open contaminated wounds could limit the EMS from giving PHAA, which is in favor of empirical prophylaxis.

**The risk for antimicrobial resistance (AMR) and culture positivity**: Prior data [7,38,39,40,41] supported the early recognition of sepsis and treatment initiation, particularly in prehospital settings, where point-of-care testing is limited. Although the UK Sepsis Trust recommended identifying “red flag” sepsis using a high index of clinical suspicion, the subjective interpretation of clinical history still contributes to variability in early sepsis recognition and adds more challenges. This need for early empiric therapy must be balanced against the harms of unnecessary or subtherapeutic broad-spectrum antibiotic exposure. In emergency care, 40% to 60% of antibiotic prescriptions may be inappropriate, contributing to AMR [42,43].

Under time pressure and diagnostic uncertainty, clinicians may appropriately initiate empiric therapy to avoid undertreatment; however, prolonged uncertainty can lead to inappropriate exposure and unfavorable consequences. Almost one third of prescriptions for non-hospitalized patients, including WHO Watch-category antibiotics, further underscore the importance of antimicrobial stewardship. The WHO AWaRe (Access, Watch, Reserve) framework classifies Watch agents as antibiotics with greater resistance potential that should be prioritized for monitoring and stewardship [44]. Subtherapeutic antibiotic concentrations may further promote resistant organisms. Integrating antimicrobial stewardship (AMS) with diagnostic stewardship may support early de-escalation, refine antimicrobial selection, and improve patient outcomes, particularly in high-turnover emergency departments [45,46].

Prehospital antibiotic administration can significantly suppress blood-culture yield, impacting downstream pathogen identification and antimicrobial stewardship. A single dose of antibiotics before culture collection could rapidly sterilize the bloodstream, leading to false-negative results [47,48]. Moreover, the effect of prior receipt of antibiotics on the pathogen distribution is specimen-dependent. However, these abovementioned confounders remain understudied in patients who received PHAA on the way to the hospital.

Future studies should move beyond the binary question and instead focus on which patients within which EMS systems derive net benefit. RCTs should enrich high-risk phenotypes, use standardized prehospital recognition criteria, report appropriateness of antimicrobial selection, capture microbiological yield, and incorporate stewardship-oriented endpoints alongside mortality. After completing this analysis, the 2026 guideline was released, recommending that PHAA be implemented if a structured process is in place to screen for sepsis (preferably “not to screen”) during transport to the hospital. This is selectively applied to adults with definite or probable sepsis and hypotension and who have an anticipated time to in-hospital medical evaluation of one hour or more [37]. Introducing the EMS point-of-care lab test could be helpful for screening during transportation.

Based on the current review, PHAA may not yet be considered a universal component of sepsis care for all patients with suspected infection. Their use appears most justifiable as a targeted strategy within well-developed EMS systems, particularly for carefully selected high-risk patients, and meaningful treatment delay is expected.

## 4. Limitations

1. Substantial clinical heterogeneity existed across studies regarding patient populations, diagnostic criteria, EMS systems, and antibiotic regimens. Certainty of evidence across outcomes was rated as very low. Even within studies of sepsis, the population is intrinsically heterogeneous. In the prehospital setting, this may be even more pronounced, because identifying “suspected infection” or “suspected sepsis” inevitably includes an element of clinical judgement. Although physiological parameters and severity scores can be standardized to some extent, clinical suspicion of infection may vary substantially between EMS systems, clinicians, countries, and available diagnostic resources. As a result, patients classified as “suspected sepsis” in one study may be quite different from those included in another.
2. Some continuous outcomes required converting nonparametric summaries (e.g., medians and interquartile ranges) to estimated means and standard deviations to allow for quantitative synthesis, which may introduce additional uncertainty, particularly for skewed outcomes. The affected studies and their originally reported values are listed in the Appendix A Table A3.
3. Meta-regression analyses were not performed, because the number of included studies was insufficient to reliably evaluate potential effect modifiers.
4. The assessment of publication bias was limited because Egger’s test is underpowered when few studies (n < 10) are available for analysis.
5. Most included studies were conducted in high-income countries with established EMS systems and protocols. Although this supports the operational feasibility of PHAA, it may limit generalizability to lower-resource settings, where prehospital infrastructure differs.
6. The absence of reported adverse events should not be interpreted as proof of safety.
7. Clinically meaningful harms—such as inappropriate exposure in non-infected patients, delayed recognition of alternative diagnoses, antimicrobial selection pressure, and interference with microbiological yield—are unlikely to be captured reliably in small trials or retrospective datasets. Safety outcomes were inconsistently reported. Randomized and feasibility trials that prespecified adverse event monitoring reported no serious adverse events attributable to PHAA, with only rare, mild allergic reactions observed [16]. Most observational studies did not report safety outcomes.
8. The heterogeneity is present in the indications and populations. Although sensitivity analyses were undertaken, this heterogeneity limits the extent to which the pooled estimates can be interpreted.
9. Observational studies are inherently vulnerable to confounding by indication and by system capability.
10. Patients selected for prehospital antibiotics may differ systematically in illness severity, clinician concern, transport pathway, and access to advanced prehospital resources, all of which may independently influence outcome, independently of the antibiotic timing. The pooled mortality signal should be interpreted as hypothesis-generating rather than practice-defining. Another important source of heterogeneity is timing. The relevant question is probably not simply whether antibiotics were given “prehospital” or “in hospital”, but how much treatment time was gained. A patient receiving antibiotics 10–15 min before arrival at an ED is very different from a patient in a healthcare system with prolonged transport or delayed hospital access, where prehospital administration could advance treatment by one or more hours [49,50].
11. Pooled estimates were calculated from crude, unadjusted event counts, as adjusted estimates were reported inconsistently across observational studies—using different effect measures and covariate sets—and were unavailable in two studies. Combined crude estimates should therefore be interpreted cautiously, particularly where adjustment altered individual study associations [19,26,29]
12. There was no study found in this review on the cost-effectiveness of PHAA, prehospital antibiotic type, blood culture, and infection severity.

## 5. Method

### 5.1. Study Design and Protocol

This systematic review and meta-analysis were conducted in accordance with the PRISMA 2020 guidelines [51]. The review protocol was developed a priori and registered with the International Prospective Register of Systematic Reviews (PROSPERO) with CRD number 420251169674 [52]. The protocol prespecified the study objectives, eligibility criteria, outcomes of interest, and analytical methods prior to the initiation of study selection.

### 5.2. Eligibility Criteria and Study Selection (PICO)

The population primarily comprises adult patients managed in the prehospital setting by the EMS, including both medical conditions and traumatic injuries, with suspected infection or sepsis. Adult and mixed-age population studies were eligible, whereas studies including only the pediatric population were excluded. The intervention was PHAA in the prehospital setting (EMS). The comparator consisted of patients who did not receive antibiotics prior to hospital arrival. The outcomes of interest included short-term (28 or 30 days) and long-term (90 days) mortality, HLOS, and ICU admission. Infection type and severity, blood-culture positivity, and time to antibiotic administration were analyzed whenever available.

Because prehospital antibiotic use may serve different clinical purposes across settings, we anticipated including distinct populations: patients with suspected sepsis or septic shock, patients with invasive meningococcal disease (IMD), and trauma patients receiving prophylactic antibiotics for open wounds. This distinction was considered clinically important a priori and informed both the interpretation of findings and the sensitivity analyses. We included randomized controlled trials (RCTs) and observational studies published in English between database inception and the final search date (January 2026). We excluded animal studies, case reports, case series, systematic reviews, editorials, conference abstracts, studies evaluating non-systemic antibiotics, and studies involving only pediatric patients. Three reviewers (IA, ALM, and MK) independently screened titles and abstracts, followed by full-text assessment of potentially eligible studies. Disagreements were resolved through discussion and consensus, with consultation from another reviewer (AE) when necessary.

### 5.3. Information Sources, Search Strategy, and Data Extraction

A comprehensive literature search was conducted in PubMed/MEDLINE, Embase, Cochrane CENTRAL, and Scopus databases. The search combined controlled vocabulary (e.g., MeSH and Emtree terms) and free-text keywords related to prehospital care, EMS, systemic antibiotics, trauma, and sepsis. No date restrictions were applied. The full search strategies are provided in the Supplementary Materials. Two reviewers independently extracted data using a standardized, pre-piloted electronic form in Microsoft Excel (Microsoft Corp., Redmond, WA, USA). Extracted data included study design and setting, participant characteristics, clinical indications, details of prehospital antibiotic exposure and comparators, funding sources, and reported outcomes. Quantitative data for meta-analysis, including effect estimates and measures of variance, were collected. Discrepancies were also resolved through discussion and consensus.

### 5.4. Risk of Bias Assessment and Certainty of Evidence

Risk of bias was assessed independently by three reviewers. For RCTs, the Cochrane Risk of Bias 2 (RoB2) tool was used. For observational studies, the Newcastle–Ottawa Scale (NOS) was applied. Disagreements were resolved by a consultation with a fourth reviewer. The certainty of evidence was evaluated using the Grading of Recommendations, Assessment, Development, and Evaluation (GRADE) approach.

### 5.5. Meta-Analysis

A meta-analysis was performed when at least two studies reported comparable outcomes and effect measures. Studies involving trauma populations were excluded from all pooled meta-analytic estimates, as trauma patients differ fundamentally from medical patients in injury mechanism, baseline physiological status, and clinical management pathway, thus limiting the validity of pooling effect estimates across these distinct populations. Such studies were retained in the systematic review narrative. Substantial clinical heterogeneity was anticipated, including differences in EMS protocols, routes and timing of antibiotic administration, and infection type (e.g., sepsis vs. invasive meningococcal disease). Given these variations in care settings, patient populations, and study methodologies, pooled estimates were calculated using random-effects models (DerSimonian–Laird). Statistical heterogeneity was assessed using the I^2^ statistic, with values of approximately 25%, 50%, and 75% representing low, moderate, and high heterogeneity, respectively. The anticipated clinical and methodological diversity across studies informed the subgroup analyses.

For dichotomous outcomes, pooled risk ratios (RRs) with 95% confidence intervals (CIs) were calculated. When mortality was reported at different time points, short-term mortality outcomes were preferentially grouped when judged to reflect the same acute illness episode (in-hospital mortality). Ninety-day mortality (long-term) was examined separately in sensitivity analyses because it may be influenced by post-acute factors beyond the immediate effect of prehospital antibiotic timing. For continuous outcomes measured on the same scale, pooled mean differences (MDs) with 95% CIs were estimated. When continuous data were reported as medians with interquartile ranges or ranges, means and standard deviations were estimated using the method described by Wan et al. This enabled their inclusion in quantitative synthesis [53].

Sensitivity analyses were conducted sequentially by excluding predefined groups of studies, including non-randomized, studies with mixed-age populations, studies involving IMD, and studies with a higher risk of bias. These analyses evaluated the robustness of pooled estimates. Given the predominance of observational data and the potential for residual confounding, these analyses also aimed to assess the stability of the observed associations. Publication bias was assessed using funnel plots and Egger’s test when sufficient studies were available. All statistical analyses were performed using R software (version 4.4.2; R Foundation for Statistical Computing, Vienna, Austria).

## 6. Conclusions

PHAA was not associated with a significant reduction in short-term mortality in pooled medical studies overall, though a significant reduction was observed when restricted to adult sepsis/septic shock population, a finding that should be interpreted as exploratory. No mortality benefit was found in pooled RCTs, mixed-age population studies, or long-term mortality. Randomized evidence does not support a mortality benefit from PHAA, while pooled analyses dominated by observational data suggested a possible benefit, reaching significance in the adult sepsis/septic shock subgroup—a finding that should be interpreted as exploratory. This discordance between randomized and observational evidence likely reflects confounding by indication, patient selection, and differences in EMS systems of care. Data on antibiotic type, prehospital blood culture, and cost-effectiveness studies are lacking. The certainty of the evidence remains very low; therefore, RCTs are warranted.

## Figures and Tables

**Table 1:** PICO.

Study ID	Center Type	Population Type	Antibiotic Class (Intervention)	Comparator	Primary Outcomes	Secondary Outcomes
Alam et al. [16] PHANTASi trial	Multicenter	Suspected sepsis	Ceftriaxone	Standard care (fluid resuscitation + supplemental oxygen)	28-day mortality—RR 0.95 (95% CI 0.74–1.24), not significant	Misdiagnoses: 1% vs. 2% (descriptive only); 90-day mortality: 12% vs. 12%, RR 0.98 (0.80–1.21), *p* = 0.87; HLOS: median 6 vs. 6 days, *p* = 0.12; ICU admission: 10% vs. 9%, RR 1.17 (0.92–1.49), *p* = 0.19; ICU LOS: median 4 vs. 3 days, *p* = 0.28; 28-day readmission: 7% vs. 10%, *p* = 0.0004, significant reduction; QoL (SF-36): physical *p* = 0.06, mental *p* = 0.46, neither significant;
Cudini et al. [24] PASS trial	Multicenter	Suspected sepsis	Ceftriaxone	No antibiotics before arrival	Time to antibiotics: median 42 vs. 150 min, difference 108 min (95% CI 34–170), *p* < 0.01, significant	eed for ICU: 42.9% vs. 42.9%, no difference; hospital LOS: 5.4 vs. 4.3 days; in-hospital mortality: 28.6% vs. 28.6%, no difference; Total blood-culture positivity: 42% (13/31);
Jones et al. [25] PhRASe trial	Single center	Severe sepsis	Cefotaxime	Standard care: supplemental oxygen, fluid bolus, pre-alert hospital in severe cases	Feasibility—all progression criteria met	90-day mortality: 33.9% vs. 21.2%, OR 1.9 (95% CI 0.82–4.5), *p* = 0.13, not significant; ICU admissions: 8.1% vs. 1.9%, OR 4.5 (0.51–39.6), *p* = 0.14, not significant; ED attendances: mean diff. −0.2, *p* = 0.1, not significant; hospital admissions: mean diff. 1.8 (1.0–2.5), *p* < 0.05, significant increase; bed days: *p* = 0.9, not significant; quality of care score: *p* = 1.0, not significant; SF-12 physical/mental: *p* = 0.5 both, not significant
Jouffroy et al. [27]	Multicenter	Severe sepsis or septic shock	Cefotaxime, Ceftriaxone	No antibiotics before arrival	30-day mortality: HR 0.56 (95% CI 0.35–0.89), *p* = 0.016, significant reduction	Hospital LOS: 7 vs. 17 days, *p* < 0.001; SOFA/SAPS2 also differed, *p* < 0.001 with intervention group having higher values—reported as a baseline/severity comparison between groups, not a declared study outcome
Kotnarin et al. [19]	Single center	Sepsis	Ceftriaxone	Standard care (fluid resuscitation)	In-hospital mortality: 32.2% vs. 47.8%; adjusted OR 0.30 (95% CI 0.11–0.82), *p* = 0.018, significant reduction	Hospital LOS: 9.5 vs. 8.0 days, *p* = 0.357, not significant; ICU admission: 7.8% vs. 6.7%, OR 1.18 (0.38–3.66), *p* = 0.774, not significant; ICU LOS: 7.0 vs. 13.5 days, *p* = 0.290, not significant
Martel et al. [28]	Single center	Sepsis	Ceftriaxone, cefepime, vancomycin	Standard care (fluid resuscitation)	In-hospital mortality: 8.5% vs. 25.5%, *p* = 0.01, significant reduction	Hospital LOS: 11.85 vs. 10.62 days, *p* = 0.66, not significant; ICU LOS: 2.51 vs. 5.18 days, *p* = 0.03, significant reduction; ventilator days: 1.91 vs. 2.48, *p* = 0.62, not significant; blood products: 0.28 vs. 1.10, *p* < 0.001, significant reduction; blood-culture contamination: 9.8% (EMS) vs. 1.9% (nursing), *p* = 0.16, not significant; vasopressor use: *p* = 0.09, not significant
Nørgård et al. [29]	Multicenter	Meningococcal disease	Penicillin G	No prehospital antibiotics	Case fatality: 11.7% vs. 6.5%; adjusted OR 2.4 (95% CI 1.0–5.6), increased case fatality with PHAT	Oral-antibiotic subgroup: 0/27 deaths (descriptive); serogroup B: adjusted OR 2.6 (0.8–8.3), increased case fatality; other serogroups: OR 0.4 (0.0–3.7), not increased
Cabellos et al. [26]	Single center	Invasive meningococcal disease	Penicillin, ampicillin, cephalosporin, amoxicillin, macrolides, beta-lactams	No antibiotics before arrival	Total mortality: 0.8% vs. 8.0%, *p* = 0.003; multivariate OR 0.108 (0.013–0.882), *p* = 0.038, protective; not significant after propensity-score adjustment, OR 0.173 (0.021–1.423), *p* = 0.103	Mechanical ventilation 48(13%) vs. 6(5%) *p* = 0.015; Arthritis 36(9%) vs. 4(3%) *p* = 0.025; DIC 62(16%) vs. 11(9%) *p* = 0.039
Karp et al. [23]	Multicenter	Open wound	Cefazolin: 60.5%, ceftriaxone: 9.9%; amoxicillin: 8.2%; clindamycin: 1.6%	No antibiotics before arrival	Survival to hospital discharge: 97.4% vs. 96.0%, *p* < 0.001, modestly higher	Associations with PHAT receipt: non-NATO affiliation OR 4.65 (1.0–20.8); tachycardia OR 3.4 (1.1–10.5); intubation OR 2.0 (1.1–3.8); tranexamic acid OR 5.6 (1.2–26.5)
Cunningham et al. [30]	Single center	Sepsis	Piperacillin/tazobactam (Zosyn), Ceftriaxone	Standard care with antibiotics after hospital arrival	Feasibility/safety (no allergic reactions reported; one culture contamination out of 29; high paramedic adherence)	Time to antibiotics: 36.04 vs. 220.76 min, *p* < 0.005, significant; Hospital admission: *p* = 0.286, not significant; ICU admission: *p* = 0.479, not significant; death in ICU: *p* = 0.285, not significant; ICU LOS: 6.9 vs. 3.33 days, *p* = 0.072, not significant; survival to discharge: *p* = 0.823, not significant; hospital LOS: *p* = 0.469, not significant; survival to 30 days: *p* = 0.183, not significant;
Bonnet et al. [31]	Single center	Sepsis	Ceftriaxone	No prehospital antibiotics	28-day mortality: 39.0% vs. 38.5%, HR 0.87 (95% CI 0.51–1.47), *p* = 0.592, not significant	7-day mortality: 30.0% vs. 29.2%, not significant; ICU admission: 33.0% vs. 33.8%, not significant; ED time to discharge: *p* = 0.571, not significant; time to ICU admission: *p* = 0.867, not significant; ICU LOS: *p* = 0.476, not significant; blood-culture positivity: 35.4% prehospital vs. 9.1% ED overall, *p* < 0.001, significantly higher;

NR = not reported, HLOS = hospital length of stay; ICU = intensive care unit; LOS = length of stay; TTA = time to antibiotics; QoL = quality of life; SF-36 = Short-Form 36 (health-related quality of life questionnaire); PHAT = prehospital antibiotic therapy.

**Table 2:** Demographics and risk assessment.

Study ID	Country/Region	Study Design	Total Sample Size	Intervention	Comparator	Age (Mean or Median)	Male (%)	Risk-of-Bias Tool Used	Risk-of-Bias Judgment
Alam et al. [16] PHANTASi trial	Netherlands	RCT	2672	1535	1137	Int: Mean 73 (Std 13.6); Con: 72.5 (14.1)	57.40%	RoB 2.0	High
Cudini et al. [24] PASS trial	Australia	RCT	35	14	21	Int: Median 70 (IQR 60–77); Con: 81 (72–87)	65.70%	RoB 2.0	High
Jones et al. [25] PhRASe trial	United Kingdom	RCT	114	62	52	Int: Mean 75.6 (Range 30–99); Con: 71.2 (28–97)	37.70%	RoB 2.0	Low
Jouffroy et al. [27]	France	Retrospective cohort	308	98	210	Total mean 70 (std 15)	67%	NOS	7 out of 9
Kotnarin et al. [19]	Thailand	Retrospective cohort	180	90	90	Total: Mean 71.6 (Std 15.7)	42.80%	NOS	8 out of 9
Martel et al. [28]	United States	Retrospective cohort	345	47	298	Int: Median 77.5; Con: 68	53%	NOS	6 out of 9
Nørgård et al. [29]	Denmark	Retrospective cohort	479	77	402	Int: Median 9 (IQR 3–17); Con: 7 (2–17)	NR	NOS	9 out of 9
Cabellos et al. [26]	Barcelona	Retrospective cohort	509	125	384	Total Median: 19 (IQR 14–50)	39%	NOS	7 out of 9
Karp et al. [23]	United States	Retrospective cohort	18,276	2384	15,892	Int: Median 25; Con: 25	98.20%	NOS	7 out of 9
Cunningham et al. [30]	United States	Prospective + historical control	63	29	34	NR	NR	NOS	7 out of 9
Bonnet et al. [30]	Switzerland	Retrospective cohort	165	100	65	Int. Mean 77.6 (std 13.7); Con. 67.1 (19.3)	60.60%	NOS	8 out of 9

Int = intervention, Con = control, NR = Not reported, RCT = randomized controlled trial, NOS = Newcastle–Ottawa scale, RoB2 = Risk of Bias 2.

**Figure A1:**
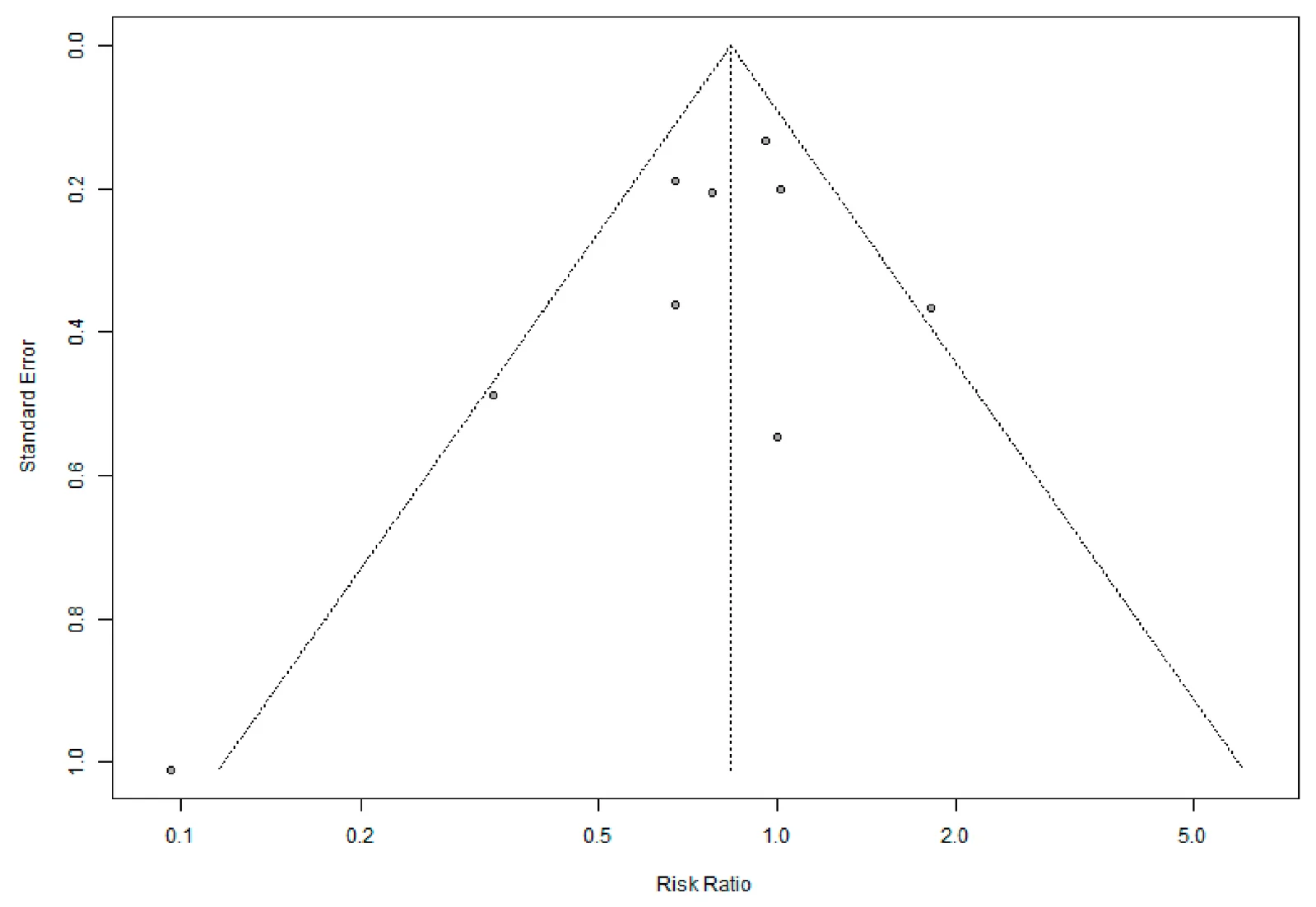
Funnel plot for short-term mortality, including medical cases only. Egger’s *p*-value: 0.26.

**Figure A2:**
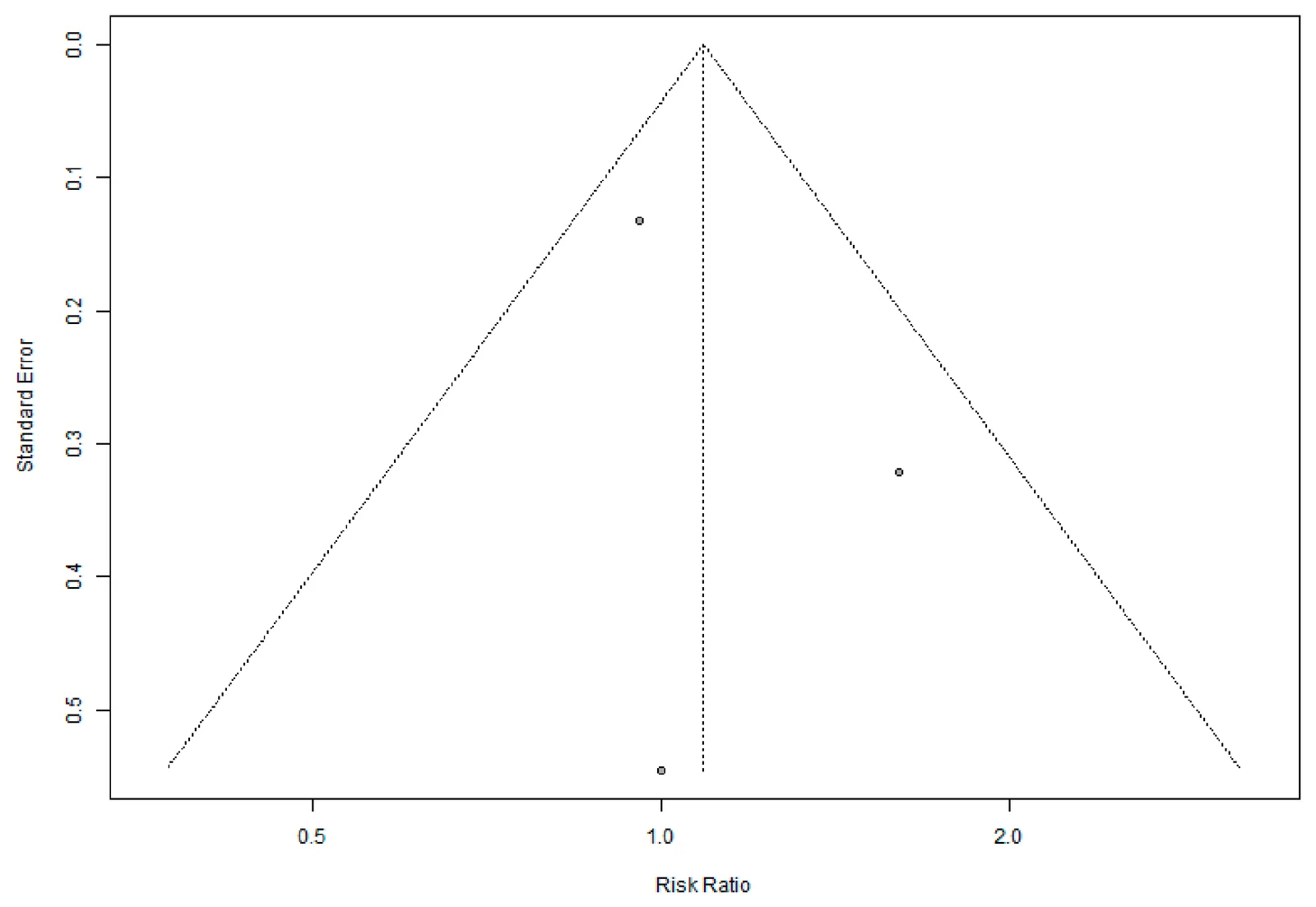
Funnel plot for total mortality for RCT only. Egger’s *p*-value: 0.60.

**Figure A3:**
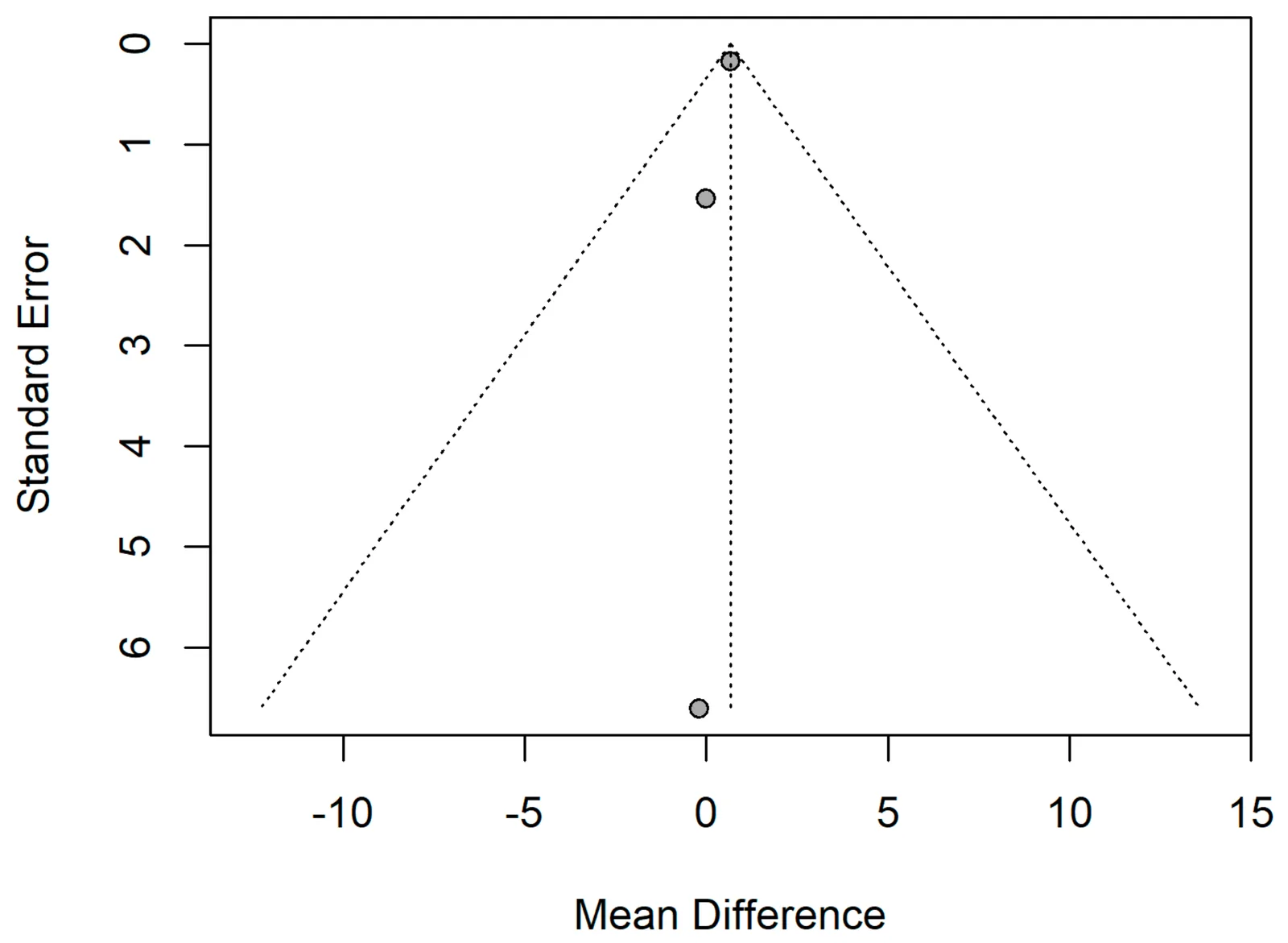
Funnel plot for hospital length of stay from all RCTs. Egger’s test *p*-value: 0.58.

**Figure A4:**
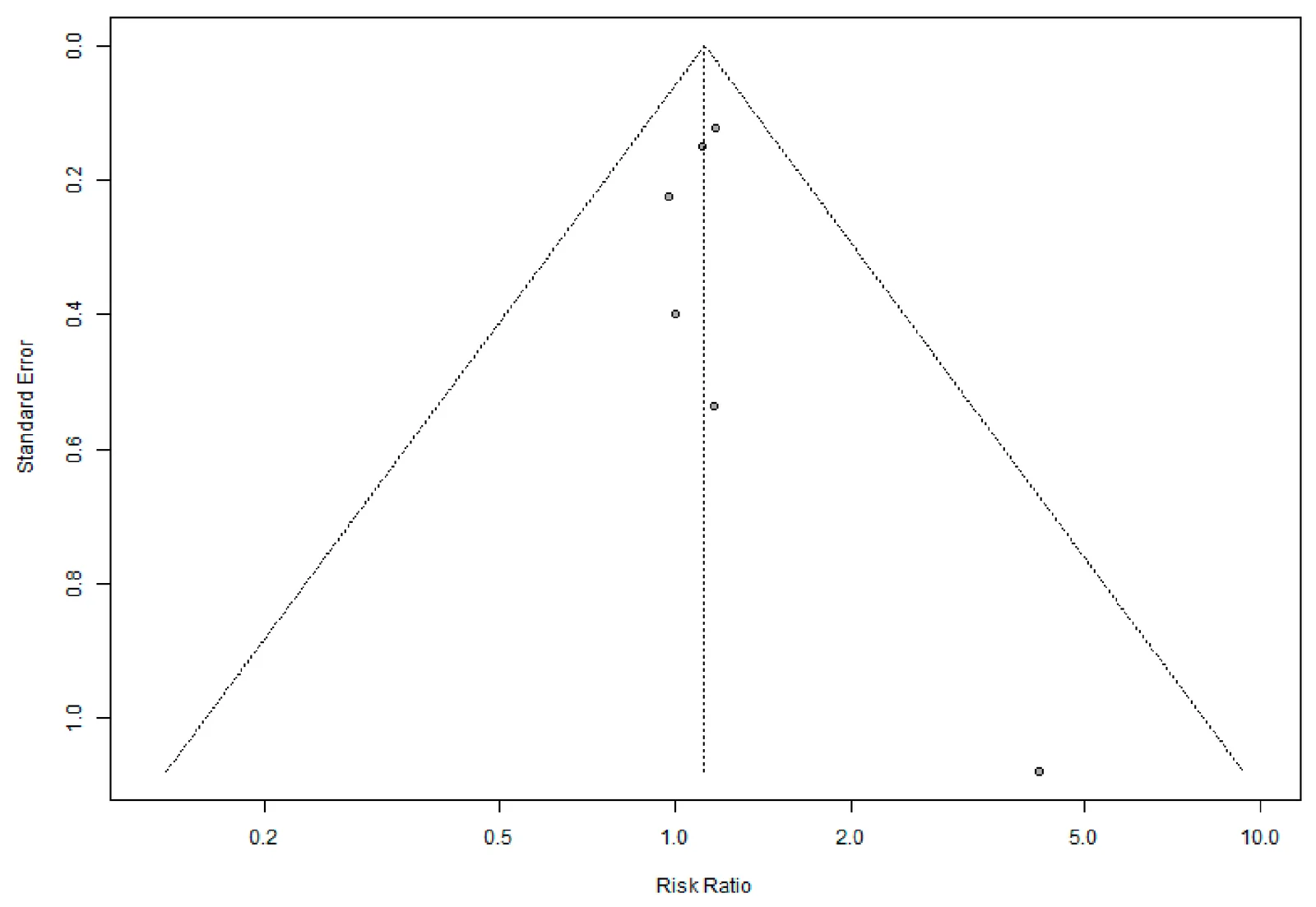
Funnel plot for ICU admissions. Egger’s *p*-value: 0.52.

**Figure A5:**
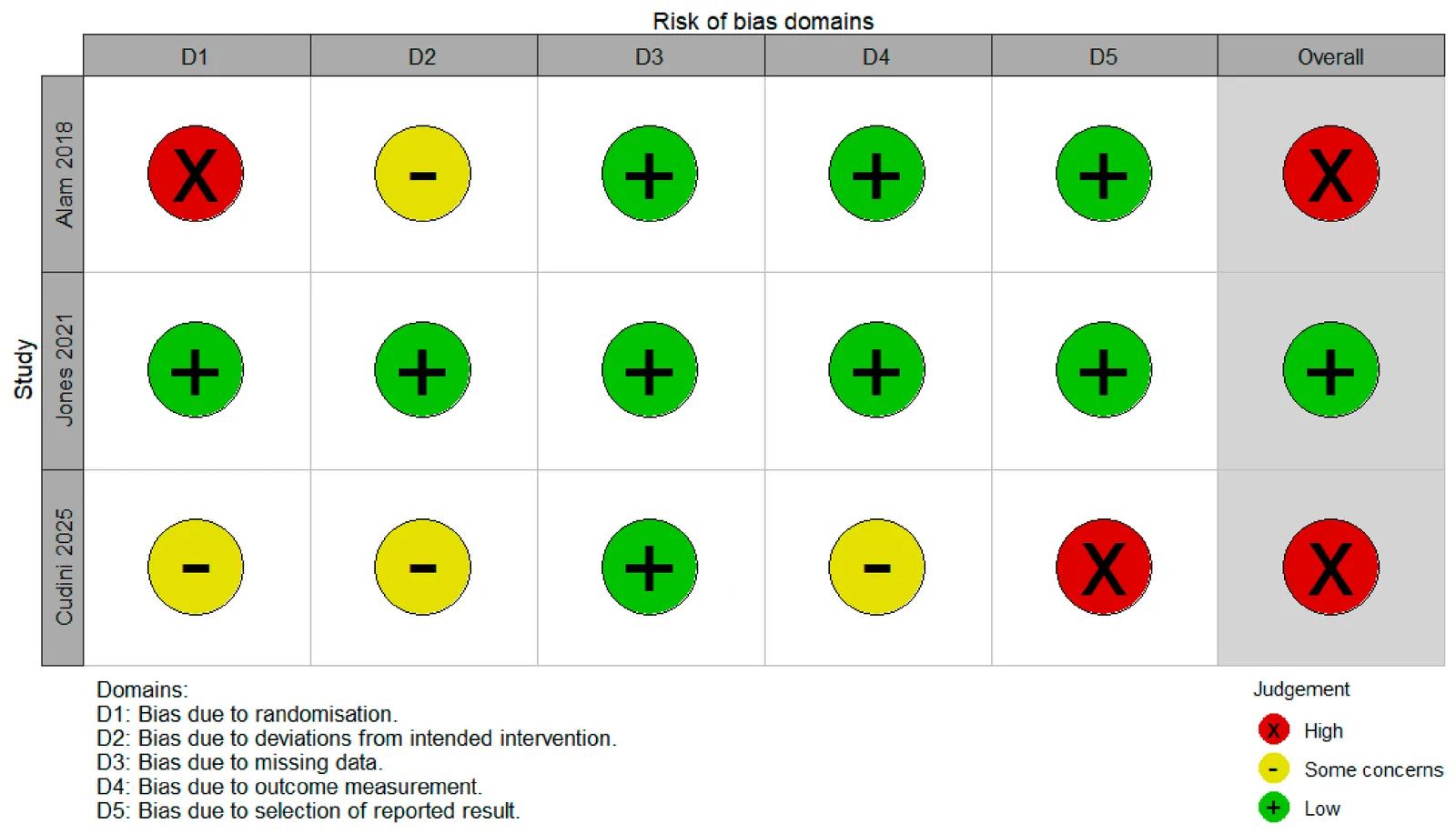
Cochrane RoB2 analysis of RCTs.

## Data Availability

No new data were created or analyzed in this study.

## Supplementary Materials

The following supporting information can be downloaded at https://www.mdpi.com/article/10.3390/antibiotics15090855/s1, File S1: PRISMA checklist.

## Abbreviations

PHAA	prehospital antibiotics administration
RCT	randomized controlled trial
EMS	emergency medical services
ED	emergency department
HLOS	hospital length of stay
ICU	intensive care unit

## Appendix A

Cochrane RoB2 analysis of RCTs and funnel plots, and Egger’s tests and GRADE tool.

**Table A1:** Time-to-antibiotic reference points by study.

Study	Temporal Reference Point	PHAA Group	Comparator	Notes
Alam et al. [16] (PHANTASi)	ED arrival	26 min before ED arrival	70 min after ED arrival	Anchored to hospital arrival, not scene contact
Cudini et al. [24] (PASS)	Paramedic scene arrival	42 min	150 min	Anchored to scene arrival
Cunningham et al. [30]	9-1-1 call receipt	36.04 min	220.7 min	Anchored to emergency call
Kotnarin et al. [19]	Not clearly defined	16.0 ± 7.4 min	50.9 ± 29.4 min	Reference point not specified in source study

**Table A2:** GRADE analysis.

Outcome (No. of Studies; Design)	Risk of Bias	Inconsistency	Indirectness	Imprecision	Publication Bias	Relative Effect (95% CI)	Certainty
Short-term mortality (9 studies; RCTs + observational)	* Serious—observational dominance; high-RoB RCTs	* Serious—discordant RCT vs. observational effects	No downgrade—appropriate population and outcome	No downgrade—CI excludes no effect	No downgrade—no clear asymmetry	RR 0.84 (0.68–1.02)	⬤◯◯◯ Very low
Hospital length of stay (7 studies; RCTs + observational)	* Serious—high-RoB RCT; observational confounding	* Serious—variable direction and magnitude	No downgrade—outcome definition appropriate	* Serious—wide CIs; small, clinically trivial effects	No downgrade—no signal detected	MD −1.79 (−6.05–2.47)	⬤◯◯◯ Very low
ICU admission (6 studies; RCTs + observational)	Serious—observational data; residual confounding	No downgrade—directionally consistent null effect	No downgrade—appropriate population and outcome	* Serious—low event rates; wide CIs	No downgrade—no signal detected	RR 1.12 (0.95–1.33)	⬤◯◯◯ Very low

**Table A3:** Nonparametric conversions.

Study	Original HLOS Reporting	Conversion Needed
Alam et al. [16] (PHANTASi)	Median 6 [IQR 3–9] vs. 6 [IQR 4–10] days	Full Wan et al. median/IQR → mean/SD
Cudini et al. [24] (PASS)	Median 5.4 [IQR 2.2–7.5] vs. 4.3 [IQR 2.5–8.3] days	Full Wan et al. median/IQR → mean/SD
Jouffroy et al. [27]	Median 10 [IQR 6–20] days (in-hospital LOS)	Full Wan et al. median/IQR → mean/SD
Kotnarin et al. [19]	Median 9.5 days (IQR 5.0–16.0)	Full Wan et al. median/IQR → mean/SD
Martel et al. [28]	Mean 12.85 days (95% CI 6.74–18.96) vs. 10.62 (95% CI 9.12–18.96)	Mean directly reported; SD estimated from 95% CI
Jones et al. [25]	Mean 14.2 days (95% CI 8.8–19.5) vs. 14.4 (95% CI 7.3–21.5)—median also reported separately (5 vs. 4)	Mean directly reported; SD estimated from 95% CI
Cunningham et al. [30]	Mean 8.36 days vs. 8.88 days; median 6 vs. 6; range only, no SD/IQR given	Mean directly reported; SD estimated from range
